# Identification of Primo-Vascular System in Abdominal Subcutaneous Tissue Layer of Rats

**DOI:** 10.1155/2015/751937

**Published:** 2015-08-25

**Authors:** Chae Jeong Lim, So Yeong Lee, Pan Dong Ryu

**Affiliations:** Laboratory of Veterinary Pharmacology, College of Veterinary Medicine and Research Institute for Veterinary Science, Seoul National University, Seoul 151-742, Republic of Korea

## Abstract

The primo-vascular system (PVS) is a novel network identified in various animal tissues. However, the PVS in subcutaneous tissue has not been well identified. Here, we examined the putative PVS on the surface of abdominal subcutaneous tissue in rats. Hemacolor staining revealed dark blue threadlike structures consisting of nodes and vessels, which were frequently observed bundled with blood vessels. The structure was filled with various immune cells including mast cells and WBCs. In the structure, there were inner spaces (20–60 *µ*m) with low cellularity. Electron microscopy revealed a bundle structure and typical cytology common with the well-established organ surface PVS, which were different from those of the lymphatic vessel. Among several subcutaneous (sc) PVS tissues identified on the rat abdominal space, the most outstanding was the scPVS aligned along the ventral midline. The distribution pattern of nodes and vessels in the scPVS closely resembled that of the conception vessel meridian and its acupoints. In conclusion, our results newly revealed that the PVS is present in the abdominal subcutaneous tissue layer and indicate that the scPVS tissues are closely correlated with acupuncture meridians. Our findings will help to characterize the PVS in the other superficial tissues and its physiological roles.

## 1. Introduction

The primo-vascular system (PVS) is a novel vascular network that was first reported in the 1960s by Kim, who claimed that the tissue corresponded with the acupuncture meridians [1]. This tissue was therefore named “Bonghan tissue” after its discoverer. More recently, the tissue has been reidentified by Dr. Soh and colleagues [2]; it was subsequently renamed the “primo-vascular system” at an international conference in Jecheon, South Korea [3]. The PVS is composed of primo vessels (PVs, or Bonghan ducts) that connect primo nodes (PNs, or Bonghan corpuscles), which are relatively thicker than the PVs [2]. The PVS tissue has been identified in various sites, such as internal organs [4–7], brain ventricles [8], and blood and lymphatic vessels [9–11] in several animal species [12]. Various techniques have been used to visualize the PVS, including trypan blue, through which many important anatomical features of the PVS have been elucidated [2], and Hemacolor utilized in recent research [6].

PVS studies in the past 10 years have identified the following hallmarks of PVS. First, the PVS primarily consists of vessel parts (PVs) and node parts (PNs), and the vessels are often subbranched from each node [2, 4, 5]. Second, rod-shaped nuclei (revealed by DNA-specific staining) are linearly aligned along the longitudinal axis of the PVS [5, 9, 11]. Third, the PVS contains various immune cells, including mast cells (MCs) and white blood cells (WBCs), such as eosinophils, neutrophils, and lymphocytes, and it has a unique cellular composition [6, 13, 14] and a high number of MCs and WBCs. Fourth, the PVS is composed of a bundle of several small subducts or ductules (10–50 *μ*m) containing immune cells [6, 13, 14]. This bundle structure is morphologically different from that of a lymphatic vessel, which only has a single lumen [5, 13].

Based on Kim's claim in the 1960s that the PVS is a reality of acupuncture meridians [1], more recent research has sought to identify the PVS in the skin or hypodermis, where the putative acupoints are located. For example, in 2009, Ogay et al. [15] showed a series of blood plexuses at the putative acupoints along the left and right kidney meridian lines in the abdominal skin of rats using a diffusive light illumination technique. In 2010, Lee and Soh [16] reported a trypan blue-stained structure along the skin skeletal muscles in the hypodermal layer of a rat. In these studies, the locations of the putative PVS tissue appeared to correspond to the acupuncture meridians in the subcutaneous area. However, clear evidence that these putative PVS tissues have the established hallmarks of the existing PVS is missing.

Therefore, in this study, the gross morphological features of putative PVS in rat abdominal subcutaneous tissue were first examined using the Hemacolor reagents, which comprise a rapid staining system that is commonly used in hematological and clinical specimens [17, 18] and has been effectively used to characterize organ surface PVS (osPVS)* in vitro* [6]. Then, we confirmed the putative PVS is distinct from the lymphatic vessel by scanning and transmitting electron microscopy. Finally, we also examined the relationships between the subcutaneous PVS (scPVS) and the acupuncture meridians of the abdominal area.

## 2. Materials and Methods

### 2.1. Animal Preparation

Male Sprague-Dawley rats weighing 120–150 g (4-5 week; *n* = 23; Orient Bio, Gyeonggi-do, Korea) were used for this study. The rats were housed in a temperate (20–26°C), relatively humid (40–70%), light-controlled (12-hour light/dark cycle; with the light coming on at 9:00 AM) environment. They were allowed open access to water and standard rodent chow* ad libitum*. The rats were allowed to adjust to this environment for 3-4 days before the experiments were conducted. All animal experiments were conducted in accordance with the Guide for the Laboratory Animal Care Advisory Committee of Seoul National University and were approved by the Institute of Laboratory Animal Resources of Seoul National University (SNU-140926-2). All surgical procedures were performed under general anesthesia.

### 2.2. Identification of osPVS and scPVS* In Vivo*

Under deep anesthesia induced with an anesthetic cocktail (Zoletil, 25 mg/kg; xylazine, 10 mg/kg) administered intramuscularly into the right femoral regions, two types of PVS samples were collected according to the following procedures. For the first procedure, the osPVS was identified on the surface of the abdominal organs using the trypan blue or Hemacolor staining method. The Hemacolor dyes were comprised of a three-solution system (Solution 1, absolute methyl alcohol for primary fixation; Solution 2, buffered eosin with sodium for eosin staining; Solution 3, phosphate-buffered thiazine for methylene blue staining) [17, 18]. These solutions (1 mL each) were applied to the surface of the internal organs, such as the intestines and the liver, in the cut abdominal cavity for 5 s. After application of Solution 3, the dye solutions were washed out using the Krebs solution (NaCl, 120.35 mM; NaHCO_3_, 15.5 mM; glucose, 11.5 mM; KCl, 5.9 mM; CaCl_2_, 2.5 mM; NaH_2_PO_4_, 1.2 mM; and MgSO_4_, 1.2 mM) [7]. After the Hemacolor staining, the revealed osPVS tissues were immediately sampled for additional staining under a stereomicroscope. For the second procedure, scPVS tissue was identified in the subcutaneous tissue layer using Hemacolor staining. Solution 1 (1 mL) was first applied to the hypodermis region of interest for 5 s, and then Solutions 2 and 3 (1 mL each) were applied to the same region one by one. Then, Solution 3 had been applied for 5 s; the region was washed out with a 0.9% saline solution and observed under a stereomicroscope (~1,000x).

### 2.3. Staining Methods for the Identification of scPVS* In Vitro*

For the characterization of gross morphology, the identified scPVS was isolated from the abdominal hypodermis region using a microscissor (blade 5 mm) and transferred into a drop of Hank's balanced salt solution (Sigma, St. Louis, MO, USA) on a slide glass. After the sample was completely air-dried at room temperature for 1–3 min, the slide glass was immersed 10 times in and taken out of dye Solution 1 for 10 s (~1/s). Additional staining with Solutions 2 and 3 was repeated using the same process. The sample was then placed in a drop of phosphate buffer solution (pH 7.2) for 20 s, washed by shaking it 10 times in distilled water for 20 s, air-dried for 3–5 min, and finally mounted with Canada balsam (Sigma). The PVS slice preparation was performed according to the previous method [6]. To confirm the DNA and RNA contents of the scPVS, the sample was kept immersed in a 0.1% acridine orange solution for 15 min, and a digital photograph was then taken using a confocal laser-scanning microscope at the wavelength of the acridine orange [19]. To verify the MCs in the scPVS, the sample was immersed in a 1% toluidine blue solution for 3 min [20].

### 2.4. Electron Microscopy of scPVS and the Lymphatic Vessel

For the scanning electron microscopy (SEM), scPVS and lymphatic vessels were collected from the rats. The procedure for harvesting the scPVS tissue was the same as that used in the sampling by Hemacolor staining. The harvesting procedure for the lymphatic vessel was as follows: under deep anesthesia, the abdomen of the rat was incised, and the lymphatic vessel was sampled under a stereomicroscope around the kidney by reference to the previous report [21]. The scPVS attached hypodermis layer (Figure 3(a)) was isolated from the area of umbilicus on the rat abdominal middle line (Figures 2(a) and 11(a)). The sampled scPVS and lymphatic vessels were kept in 2% paraformaldehyde for 24 hr and primary fixed by Karnovsky's fixation for 2 hr. After washing them with 0.05 M sodium cacodylate buffer 3 times for a total of 10 min, the samples were postfixed by 2% osmium tetroxide (1 mL) and 0.1 M cacodylate buffer (1 mL) and washed twice using distilled water for 5 s each. Dehydration was conducted using a series of ethanol of 30%, 50%, 70%, 90%, and 100% for 10 min each, and then the samples were dried using a critical point dryer (CPD 030, BAL-TEC) for 1 hr, coated by a sputter coater (EM ACE200, Leica), and observed under a field-emission SEM (SIGMA, Carl Zeiss, UK). For transmission electron microscopy (TEM), several procedures were added to those of SEM. (1) After washing the samples with distilled water, en bloc staining was conducted with 0.5% uranyl acetate for 30 min; (2) after dehydration with ethanols, a transition procedure using propylene oxide was conducted twice for 10 min each; (3) then the samples were embedded into Spurr's resin (2 mL), sectioned, and observed under a TEM (JEM1010, JEOL, Japan).

### 2.5. PVS Cell Counting and Data Analysis

The PVS cells were counted from 21 fields (100 × 100 *μ*m) in an image of the Hemacolor staining of the PVS tissues (*n* = 7) at magnifications of 400 and 1,000x. The sizes of the PNs, PVs, and resident cells were measured using image J software (developed at the US National Institute of Health). All results are shown as mean ± standard errors, and the number of samples or cells was represented by *n*.

## 3. Results

### 3.1. Gross Morphology of the scPVS* In Vivo*

The results of the present study were obtained from the examinations of subcutaneous PVS (scPVS) tissues in 14 rats and organ-surface PVS (osPVS) tissues in 7 rats. The osPVS tissues (*n* = 17) were sampled mainly from the surfaces of the abdominal organs, including the large intestine (32%), the small intestine (38%), and the liver (17%). The scPVS tissues (*n* = 24) were sampled exclusively from the abdominal subcutaneous area. The osPVS tissues were identified without staining, whereas the scPVS tissues were identified with Hemacolor staining.

**Figure 1:**
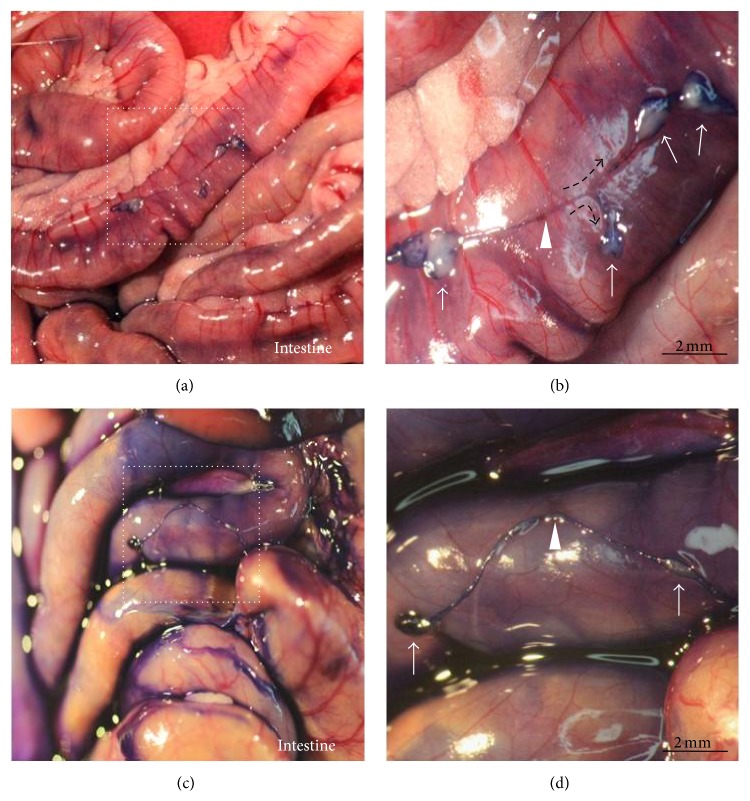
Identification of intact PVS tissue on the surface of abdominal organs in rats using Hemacolor and trypan blue staining. (a) PVS tissue on the surface of the small intestine stained by Hemacolor. (b) Magnified view of the organ surface PVS structure (square in (a)) composed of multiple-PNs (arrows), a PV (arrowhead), and a subbranching point (dotted arrows). (c) PVS tissue on the surface of the large intestine stained by trypan blue. (d) Magnified view of the organ surface PVS structure (square in (c)) composed of two PNs (arrows) and a PV (arrowhead).

Previously, it has been shown that Hemacolor staining is effective in characterizing the gross morphology of the osPVS in isolation [6]. In this study, Hemacolor dyes were directly applied to the PVS tissues on the surfaces of the abdominal organs to determine whether the osPVS could be stained* in vivo* as it is stained by trypan blue, which is a well-known dye that is used to identify PVS* in situ* [2]. The Hemacolor staining revealed that the PVS was attached to the surface of the abdominal organ. Figures 1(a) and 1(b) show representative osPVS tissue composed of primo-nodes (PNs) and primo-vessels (PVs) on the surface of the small intestines revealed by Hemacolor staining. The Hemacolor staining of the osPVS was comparable to the trypan blue staining (Figures 1(c) and 1(d)).

**Figure 2:**
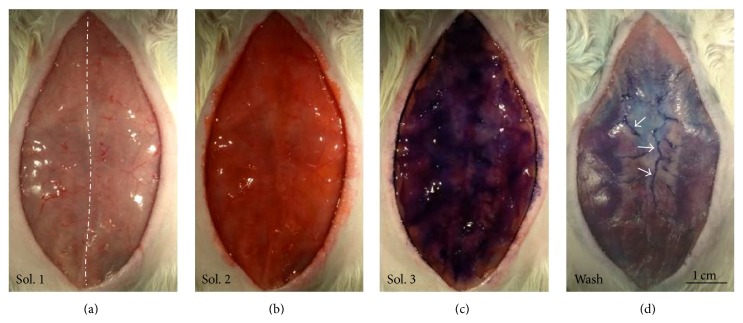
Identification of the threadlike structures in the rat abdominal subcutaneous tissue using Hemacolor staining. ((a), (b), and (c)) Appearance of the subcutaneous area after serial applications of three Hemacolor solutions: Solution 1 (methanol fixative, (a)), Solution 2 (eosin stain, (b)), and Solution 3 (methylene blue stain, (c)). Dotted line is the abdominal middle line. (d) Appearance of the subcutaneous area after wash-out of Hemacolor solutions. Note the dark blue threadlike structures (arrows). See the* Materials and Methods* sections for a description of the protocol.

**Figure 3:**
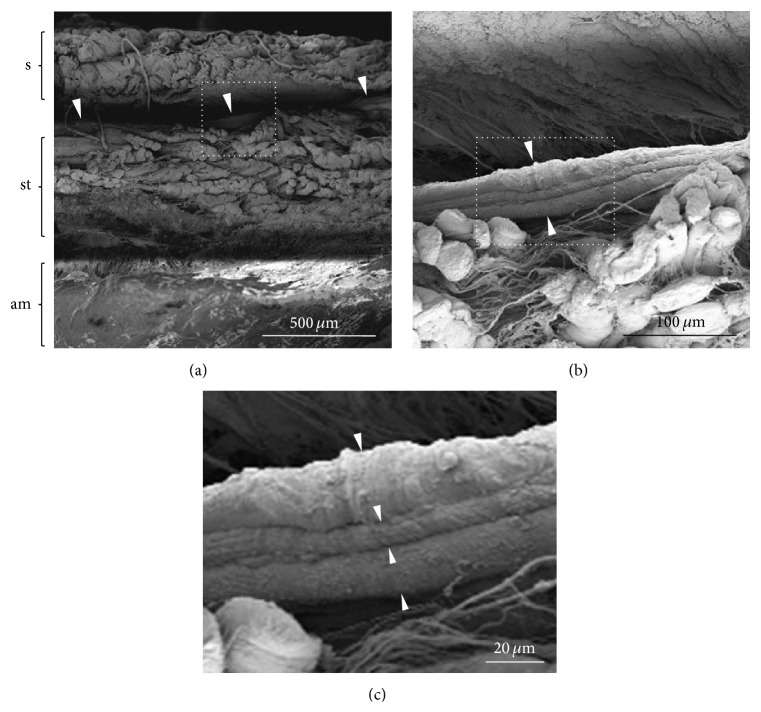
The location of the threadlike structure in the rat abdominal tissue layer revealed by scanning electron microscopy. (a) Cross-section of tissue explant including the skin (s), subcutaneous tissue layer (st), and abdominal wall muscle (am). The explant is isolated from the near area of umbilicus on the abdominal middle line. Note that the threadlike structure (arrowheads) is located between the skin and subcutaneous tissue layer. (b) Magnified view of the threadlike structure (square in (a), arrowheads) on the surface of subcutaneous tissue layer. (c) Magnified view of the threadlike structure (square in (b)) showing a bundle structure of three subducts (arrowheads). Note the rough surface of threadlike structure.

Figure 2 shows the appearance of the abdominal subcutaneous region after the applications of Solutions 1, 2, and 3 for the Hemacolor staining* in vivo*. After completion of the Hemacolor staining, dark blue threadlike structure was observed through the fascia in the stained area of the abdominal subcutaneous tissue layer (Figure 2(d)).  Figure 3 shows the anatomical location of the Hemacolor stained-dark blue threadlike structure on the surface of the subcutaneous tissue layer identified by scanning electron microscopy (SEM). This threadlike structure was comprised of a bundle of subducts or small tubes; this morphology was characteristically different from that of the lymphatic and blood vessels, which consisted of a single tube.

**Figure 4:**
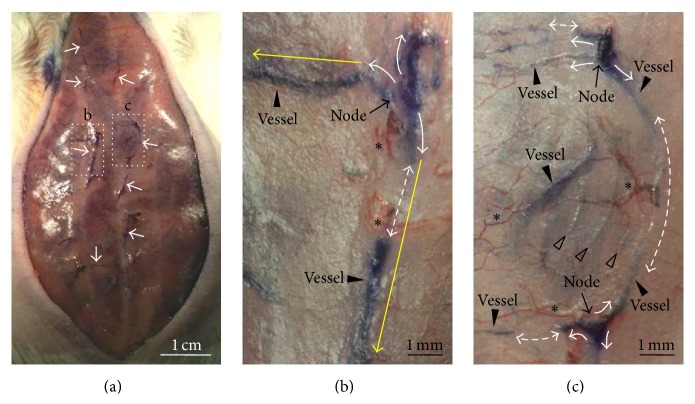
Hemacolor-stained threadlike structures in the rat abdominal subcutaneous tissue. (a) Typical example of the distribution of threadlike structures (white arrows) on the subcutaneous area stained by Hemacolor. ((b) and (c)) Threadlike structures (dotted squares marked as “b” and “c” in (a)) composed of the vessels (arrowheads) that connect the nodes (arrows). Note that the node structures are multibranched (white arrows) from the nodes, and the vessel structures are distributed either on or beneath the subcutaneous surface (two-way dotted white arrows and open arrowheads). Note that the blood vessels (BVs, asterisks) maintain their red color and that threadlike structures are aligned in either a vertical or horizontal direction (yellow arrows). Note the blood vessels around the branching points of the threadlike structures (asterisks).

Figure 4 shows a representative example of a dark blue-colored threadlike structure revealed by Hemacolor staining in the abdominal subcutaneous area. The vessels were divided into a vertically elongated type (yellow vertical arrow) along the abdominal middle line and a horizontally elongated type (yellow horizontal arrow) branched from each node form (Figure 4(b)). As shown in Figures 4(b) and 4(c), the dark blue-colored multiple-branched threadlike structures (white arrows) were distributed across the surface of the subcutaneous tissue. In addition, some vessels were also distributed beneath the surface of the subcutaneous tissue (Figures 4(b) and 4(c), two-way dotted white arrows and open arrowheads). The dark blue threadlike structures appeared to be interrupted where the structures were buried underneath the surface of the subcutaneous tissue layer. In addition, blood vessels were observed around the threadlike structures (Figures 4(b) and 4(c), asterisks). The blood vessels were not stained dark blue like the threadlike structures by Hemacolor staining; rather, they maintained their original red colors. The average size of the nodes of the threadlike structures was 0.94 ± 0.14 mm (*n* = 12), and the average thickness of the vessels was 0.21 ± 0.05 mm (*n* = 15).

### 3.2. Cytomorphology of the scPVS* In Vitro*

**Figure 5:**
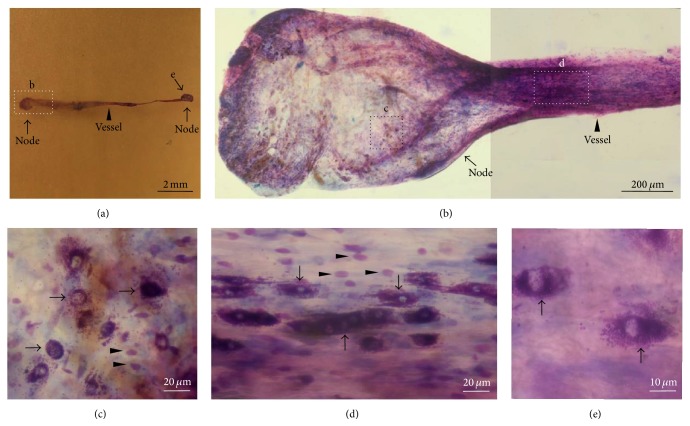
Hemacolor staining of the threadlike structure isolated from the rat abdominal subcutaneous tissue. (a) Threadlike structure isolated from the Hemacolor-stained layer of the subcutaneous tissue. (b) Typical longitudinal image of a whole threadlike structure (marked as “b” in (a)). ((c) and (d)) Two major types of cells in the threadlike structure: large granular (arrows) or small round cells (arrowheads). Note that the cells in the node (c) and in the vessel (d) are aligned differently. (e) Toluidine blue staining of the large granular cells (arrows).

**Figure 6:**
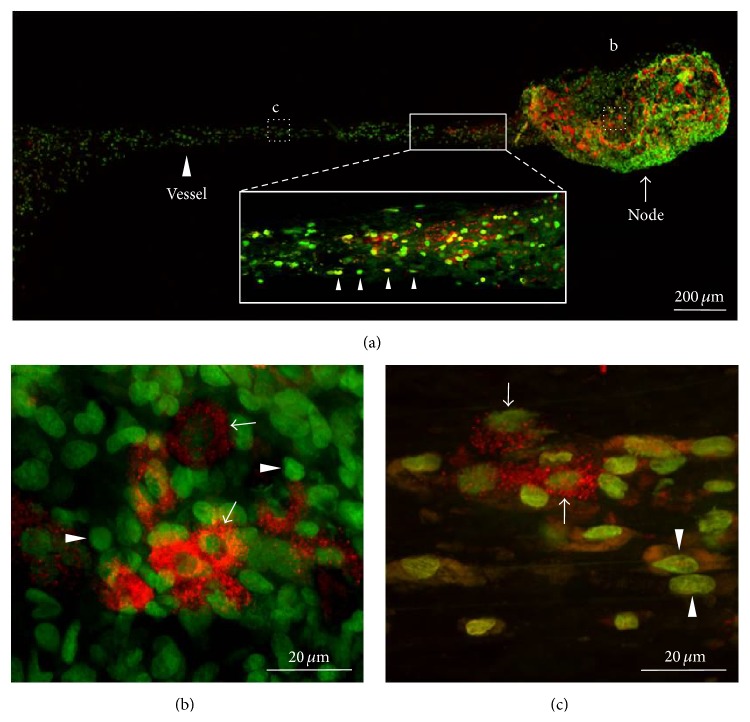
Confocal laser-scanning microscopic image of the cells in the threadlike structure stained with acridine orange. (a) Typical longitudinal image of the whole threadlike structure. Note that the cells (arrowheads in bottom inset) in the vessel are linearly aligned along the longitudinal axis of the vessel. (b) Acridine orange-stained cells in the node (marked as “b” in (a)) showing the small round cells with green nuclei (arrowheads) and large cells with green nuclei and red granules (arrows). (c) Acridine orange-stained cells in the vessel (marked as “c” in (a)). Note that the density and alignment of resident cells are different in the node (b) and in the vessel (c).

The threadlike structure was isolated and the tissue was restained* in vitro* to identify the cellular properties of the threadlike structure stained by Hemacolor.  Figure 5(a) shows a sample on the slide glass of a threadlike structure composed of nodes and vessels connected by these nodes.  Figure 5(b) is a representative stereoscopic image of the threadlike structure stained by Hemacolor. The Hemacolor-stained cells of the threadlike structure were mainly classified into two groups based on their morphologies: small round cells (~10 *μ*m) and large granular cells (10–20 *μ*m). The cells in the vessels and nodes differed in shape and distribution. The cells within a node were mostly round in shape and were dispersed unsystematically (Figure 5(c)). In contrast, the cells within a vessel were elliptical and linearly aligned along the longitudinal axis of the vessel (Figure 5(d)).  Figure 5(e) shows the image of the cells of the threadlike structure revealed by toluidine blue staining, which is widely used to stain selectively MCs [20]. In previous studies, the PVS has been found to contain a variety of immune cells, including MCs and WBCs [6, 17]; therefore, in this study, toluidine blue staining was performed. The granules within large granular cells showed the typical metachromatic staining property of toluidine blue staining, indicating that the large granular cells in the threadlike structure were MCs (Figure 5(e)) [20]. The threadlike structure was also stained with acridine orange staining to determine DNA (revealed green) and RNA (revealed red) components for the further characterization of the cellular properties of the threadlike structure [19].  Figure 6(a) illustrates the threadlike structure consisting of one node and connecting vessel stained with acridine orange. The majority of cells in the threadlike structure were stained a green color, as shown in Figure 6. The nuclei of the small round cells were stained green (Figures 6(b) and 6(c), arrowheads), whereas the large granular cells were stained green and red in the nuclei and surrounding granules, respectively (Figures 6(b) and 6(c), arrows). The shape and distribution of the acridine orange-stained cells were similar to those of the Hemacolor staining, as shown in Figure 5. Most cells in the vessel were aligned along the long axis of the vessel (Figures 6(a), arrowheads in the bottom inset, and 6(c)). In contrast, the cells in the node were randomly distributed without any clear direction (Figure 6(b)).

### 3.3. Structural Properties of the scPVS* In Vitro*

**Figure 7:**
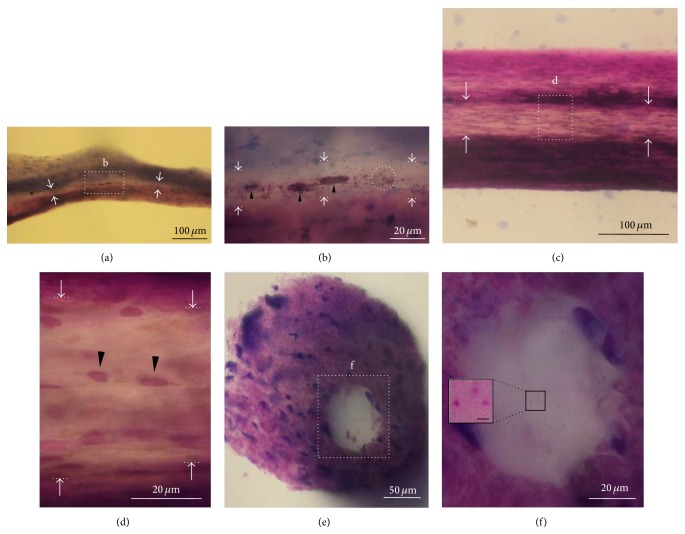
The inner space structure containing cells along the inside of the subcutaneous threadlike structure. (a) Continuous inner space (arrows) along the longitudinal axis of a threadlike structure revealed by Hemacolor staining. (b) Distribution of the cells (large granular cells, arrowheads; granules, dotted circle) in the inner space (marked as “b” in (a), 20–30 *μ*m) of the vessel. (c) Large inner space structure (> 50 *μ*m) inside the subcutaneous threadlike structure showing low cellularity revealed by Hemacolor staining. (d) Distribution of the cells (small round cells, arrowheads) in the inner space (marked as “d” in (c)) of the vessel. (e) Cross-sectional image (200 *μ*m) showing an inner space (dotted square, > 50 *μ*m) of a threadlike structure revealed by Hemacolor staining. (f) Inner space (marked as “f” in (e)) within a vessel slice containing granules (about 1 *μ*m, inset). Note that the inner space contains the resident cells of the threadlike structure.

The inner regions of the vessel of the threadlike structure revealed by Hemacolor staining appeared as white space, indicating a lower cellularity (Figure 7). The inner space was linearly continuous along the longitudinal axis of the vessel (Figures 7(a) and 7(b)). The diameters of the inner space were 20–60 *μ*m. The inner space structures contained various cells, such as large granular cells (Figure 7(b), arrowheads), granules (Figure 7(b), dotted circle), and small round cells (Figure 7(d), arrowheads).  Figure 7(c) shows a bundle-like structure revealed by Hemacolor staining as shown in Figure 3 by scanning electron microscopy. In addition, a cross-sectioned slice (200 *μ*m) of the threadlike structure was stained, and the small and large granular cells were observed in the whole threadlike structures. As shown in Figures 7(e) and 7(f), there was a sinus (~60 *μ*m) that contained granules (~1 *μ*m).

### 3.4. Comparison of the Ultrastructural Features between the scPVS and Lymphatic Vessel

**Figure 8:**
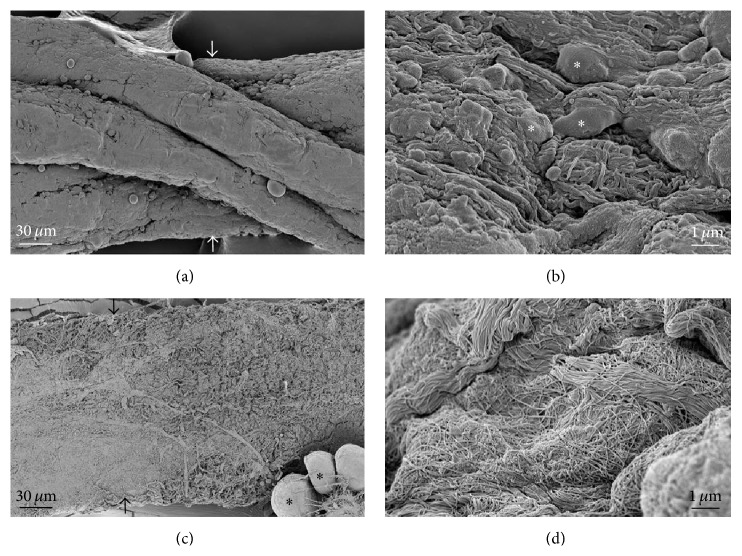
Comparison of the subcutaneous threadlike structure and lymphatic vessel using scanning electron microscopy. ((a) and (c)) Typical longitudinal images of the threadlike structure and lymphatic vessel. Asterisks are an adipocyte attached to the lymphatic vessel. ((b) and (d)) The surfaces of the threadlike structure and lymphatic vessel at higher magnification. Asterisks are the cells on the surface of the threadlike structure. Note that the threadlike structure and lymphatic vessel are different in terms of the presence or absence of the bundle structure of several subducts and cells.

**Figure 9:**
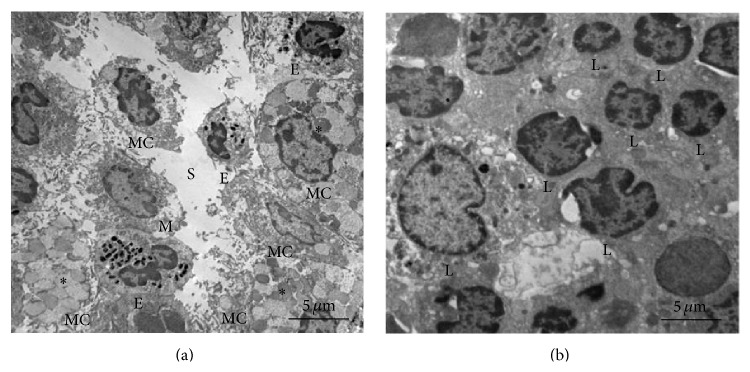
Comparison of the subcutaneous threadlike structure and lymphatic vessel using transmission electron microscopy. (a) Threadlike structure containing a sinus structure (S), mast cell (MC) and its granules (asterisks), monocyte (M), and eosinophil (E). (b) Lymphatic vessel containing mostly lymphocytes (L). Note that there is a difference in the major resident cells between the threadlike structure and the lymphatic vessel.

Electron microscopy was used to show differences between the threadlike structure and the lymphatic vessel in isolation (Figure 8). The threadlike structure was comprised of a bundle structure of several subducts (Figure 8(a)), whereas the lymphatic vessel did not appear to have bundle structure (Figure 8(c)). At a higher magnification view, we observed the round cells (Figure 8(b), asterisks) and fine fiber structures on the surface of the threadlike structure. In contrast, in the lymphatic vessel, such cells were not found and the fiber structures were more extensive than those of threadlike structure (Figures 8(b) and 8(d)).  Figure 9 shows the major resident cells of the threadlike structure and the lymphatic vessel revealed by transmission electron microscopy. As can be seen, the threadlike structure contains various immune cells, including MCs, eosinophil, and granules (Figure 9(a)), whereas the lymphatic vessel is mostly comprised of lymphocytes (Figure 9(b)). Thus, there are clear differences between the threadlike structure and lymphatic vessel in terms of the presence or absence of the bundle structures and the types of resident cells.

### 3.5. Comparison of the MC Density between the scPVS and the osPVS

**Figure 10:**
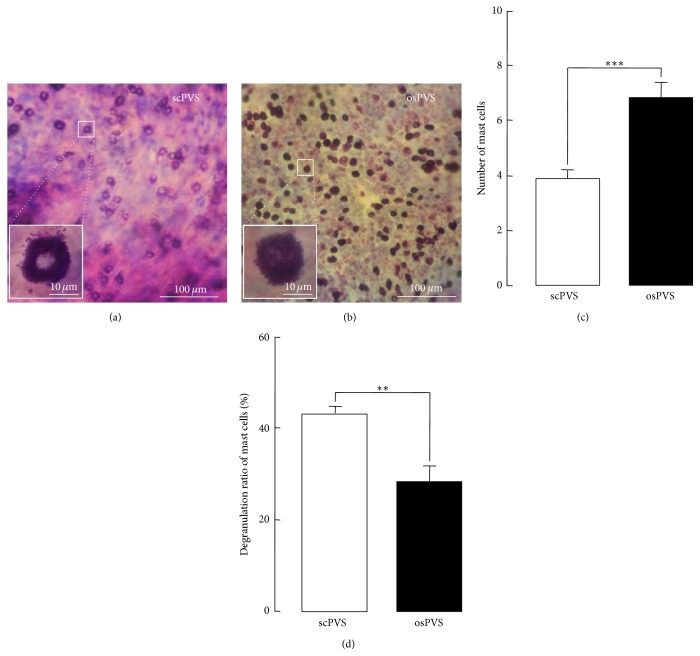
Comparison of the density and degranulation ratio of MCs identified in Hemacolor-stained scPVS and osPVS tissue. ((a) and (b)) Representative photomicrographs showing the distribution of MCs in the PNs of scPVS and osPVS stained with Hemacolor. ((c) and (d)) Summary bar graphs showing the number of MCs (c) and the degranulation ratio of these MCs in the scPVS and osPVS tissue (d). PVS cells counted from 21 fields (100 × 100 *μ*m) in an image of the Hemacolor staining of the PVS tissue (*n* = 7) at magnifications of 400 and 1,000x.

The above results regarding gross morphology, cytomorphology, and structural properties have many features in common with the well-established PVS [2, 5, 6]; thus, the subcutaneous threadlike structure, vessel, and node part were identified as the scPVS, the PV, and the PN, respectively. In addition, the large granular cells and small round cells of the threadlike structure were also identified as the MCs and WBCs of the PVS reported previously in the osPVS [6].  Figure 10 shows a comparison of the density and degranulation ratios of the MCs in the scPVS and osPVS tissues stained by Hemacolor. The cell densities of the MCs in the scPVS were lower than that of the osPVS (Figures 10(a) and 10(b)), and the degranulation ratio of the MCs in the scPVS was higher than the ratio in the osPVS (Figures 10(c) and 10(d)), which indicates that the scPVS is different from the osPVS in MC activation.

### 3.6. Relationship between the scPVS and Acupuncture Meridians

**Figure 11:**
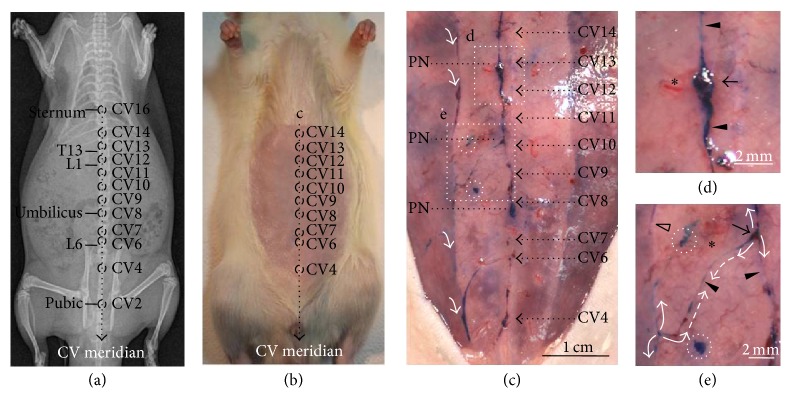
The distribution of scPVS in the abdominal subcutaneous tissue in a rat. ((a) and (b)) Locations of the conception vessel (CV) acupuncture meridian in a rat (L1, L6, and T13; lumbar 1, lumbar 6, and thoracic 13 vertebrae, resp.). (c) Typical example of the scPVS tissue (dotted square marked as “c” in (b)) on the abdominal subcutaneous tissue layer in relation to the CV meridian and the acupoints. Note other abdominal scPVS tissues away from the CV meridian line (white arrows and dotted circles). (d) scPVS corresponding to putative CV 12 and 13 (dotted square marked as “d” in (c)) comprised of a PN (arrow) and a PV (arrowheads). (e) scPVS corresponding to putative CV 9 and 10 (dotted square marked as “e” in (c)) comprised of a PN (arrow) and PVs (arrowheads). Note that there are three direction-branched PVs (white arrows) from a branching point of a PN, and two branches are connected to one vessel (dotted arrows); there is another vessel away from the vessel located at the ventral midline (open arrow). Note the blood vessels around the scPVS tissue (asterisks in (d) and (c)).

Hemacolor staining of the abdominal subcutaneous tissue revealed that there were multiple scPVS tissues over the whole abdominal space, as shown in Figures 2 and 4. Among these, the most consistently observed tissue was the scPVS tissue, which was located on the abdominal middle area (Figure 11). Since the conception vessel (CV) meridian is also known to be located at the ventral midline connecting the umbilicus to the sternum [22], the correlation between the positions of the scPVS present in the ventral midline and the CV meridian was also examined. The CV meridian and acupoints are illustrated in Figures 11(a) and 11(b). Figure 11(c) shows a typical example of scPVS tissue in the abdominal subcutaneous tissue visualized by Hemacolor staining. The most notable observation is that the scPVS is mainly distributed along the ventral midline, which consists of the vessels connected by nodes. The nodes appear to correspond to the CV acupoints (i.e., CV13, CV10, and CV8). For example, the node corresponding to CV13 appears to be enlarged and located near the blood vessel (Figure 11(d)). The node corresponding to CV10 appears to be subbranched and connected another node of the scPVS (Figure 11(e), open arrow) located in the right lateral area. A dark blue structure, scPVS, was also noted that it appeared to be mixed with bloods/plexus (Figure 11(e), arrow). Collectively, these observations indicate that the scPVS in the middle of the abdominal subcutaneous tissue layer is likely to correspond to the CV acupuncture meridian. In addition to scPVS in the ventral midline, scPVS tissue was also located laterally, at about 6 mm (Figure 11(c), dotted circles) and 9 mm from the midline (Figure 11(c), white arrows). A connection between each scPVS was also noted (Figure 11(e), dotted arrows).

## 4. Discussion

In this study, we identified the PVS in the abdominal subcutaneous tissues in rats using various staining techniques. The scPVS consisted of vessels that connect the node parts and that the vessels were frequently subbranched. In the Hemacolor-stained whole scPVS tissue in isolation, the cells of the scPVS were found to be composed of WBC-like small round cells and MC-like large granular cells. The cell distributions differed among the nodes (showing a random distribution of round-shaped cells) and the vessels (showing a linear arrangement of elliptical-shaped cells along the long axis of the vessel). Acridine orange staining showed green-stained WBC nuclei (denoting DNA) and green-stained MC nuclei with red-stained granules (denoting RNA). In addition, the inner space-channel (20–60 *μ*m) structure containing WBCs and MCs along the inside of a vessel of the scPVS was identified. Electron microcopy revealed that the threadlike structure has a bundle structure of subducts and round cells on the surface and that these were not found in the lymphatic vessel. The distribution pattern of scPVS tissues in the ventral midline was similar to the route of the CV meridian. Collectively, these findings indicate that the PVS is also present on the subcutaneous layer and that the scPVS is likely to be closely related to the acupuncture meridians in the subcutaneous tissue layer.

Compared with the unique characteristics of well-established PVS tissue, the putative scPVS of the present study had several features in common with the existing PVS, which is primarily composed of PVs and PNs that are often branched into multiple vessels [2, 4, 5]. As shown in Figure 4, the scPVS also has vessels that connect the nodes, and there are well-developed branches that form each node. The rod-shaped nuclei (10–20 *μ*m) of the existing PVS were revealed by DNA-specific fluorescent staining, including acridine orange, 4′,6-diamidino-2-phenylindole (DAPI), and the Feulgen reaction [5, 10, 11]. The scPVS revealed that the rod-shaped nuclei were aligned along the longitudinal axis of the vessel stained by acridine orange (Figure 6). The PVS is also known to contain various immune cells [6, 13, 14], and in this study, the scPVS showed many WBCs and MCs (Figures 5 and 9). In terms of the internal structure of the PVS, a bundle structure and diverse subducts (~10 or 20–50 *μ*m) that pass through a vessel or node containing the WBCs, MCs, and granules of about 1 *μ*m in diameter were noted [5, 6, 13]. Figures 7, 8, and 9 demonstrate a bundle structure, subducts, and sinus within the vessels of the long axis or a cross-section of the scPVS, including WBCs, MCs, and granules. These findings indicate that the newly identified scPVS belongs to the existing PVS group. However, there were a few different characteristics between the scPVS and the existing PVS. For example, one can differentiate the osPVS tissue from the surrounding organ tissue because it is semitransparent, freely movable, and present on the surfaces of the internal organs [4–7]. That is, it is possible to identify the osPVS without any staining. In contrast, the scPVS tissue, which is located in the interior regions as well as on the surface of the hypodermis, is not easily identified in adjacent subcutaneous tissues; thus, it is necessary to stain the hypodermis region to distinguish the scPVS from the surrounding tissue. In addition, the vessels of the scPVS are more elastic than of the osPVS, and they are often bent during the process of scPVS sampling.

The presence of enriched MCs in osPVS has been confirmed in previous studies, and they have been considered to be the major resident cells of osPVS [6, 14]. MCs have primarily been viewed as effectors of allergic/inflammatory reactions [23]. The results of the present study indicate that the scPVS also contains MCs, but they are different from those in osPVS in terms of density and their degranulation ratio, which are significant parameters that are widely used in studies on MCs. In this study, the MC density was higher in the osPVS than in the scPVS; however, the granulation ratio was higher in the scPVS than in the osPVS (Figure 10), indicating that the MCs in the scPVS are preferentially activated compared to the MCs in the osPVS. Thus, the scPVS could be more exposed to immune disorders, including physical agents, products of diverse pathogens, and many innate danger signals than osPVS. This idea is consistent with a recent hypothesis on the PVS by Stefanov et al. (2013) [24]. In the hypothesis, the external PVS such as scPVS functions as a receiving PVS that transforms the external stimuli into a type of PVS signals, whereas the internal PVS such as a osPVS functions as communicating PVS that transmits the PVS signals among different PVSs. This may explain the higher level of MC activation in the scPVS. It is well-known that MC degranulation is remarkably increased by acupuncture on the acupoints of skin tissue [25]. Thus, it is conceivable that, in this study, the MCs were more activated when being associated with the position of the CV meridian and its acupoints in the scPVS than in the osPVS.

One of the primary objectives of this work was to correlate the scPVS and acupuncture meridians, which are known to be present in the skin [22, 24]. The present study provides experimental evidences supporting the close correlation between the scPVS and meridians in the abdominal subcutaneous tissue. Firstly, the PNs revealed by Hemacolor staining showed key features of the Bonghan corpuscle. In the early 1960s, Kim reported that the Bonghan corpuscle (denoting PN) corresponding to acupuncture meridians/acupoints was present in the subcutaneous tissue layer and was connected by a bundle of blood vessels and Bonghan ducts (denoting PVs) [1, 2]. Recently, in 2009, Ogay et al. observed a series of blood vessel plexuses at the putative acupuncture points along the left and right kidney meridians in the abdominal subcutaneous tissue layer of rats by using a diffusive light illumination of the region of skin [15]. This report elucidated the morphometric scales of the blood vessel plexus at the kidney and stomach meridians, which are 4-5 mm and 2 cm from the conception vessel meridian in rats by 6–12 weeks. However, they were not able to conclude that the blood plexuses are the anatomical structures of subcutaneous Bonghan corpuscles because the PNs or PVs were not found with the bundle of the blood vessels. The findings in the present study are consistent with the Kim's claim that the scPVSs composed of PNs and PVs are frequently bundled with the blood vessels (Figures 4 and 11). This study also found that the PVS tissue in the subcutaneous layer contains MCs (Figures 5 and 9). This observation is consistent with previous studies that MCs are rich at the various acupuncture points [25, 26]. The scPVS located on the ventral midline of the abdominal space appears similar to the CV meridian. As shown in Figure 11, the distribution of the nodes, vessels, and subbranches of the scPVS is in good agreement with the known features of the CV meridian.

The Hemacolor staining mainly used in this experiment is a commercially available tool for rapid blood smears that permits good morphological judgment of cells, such as lymphocytes, monocytes, and neutrophilic granulocytes. The total process takes less than 10 min and can be done with prepared dyes without technical assistance [27]. In this study, Hemacolor staining was used as a major diagnostic tool for the PVS because it was possible to identify the PVS* in situ* as well as its cellular properties and structures. In the case of trypan blue, which is a well-known dye that is widely used to identify PVS* in situ*, its use for elucidating detailed information about the cytology and anatomical structure of PVS is somewhat limited although it is easy and simple to use [6].

One of the limitation of this study is that scPVS was not always detected as a uniform pattern or continuous threadlike structure after Hemacolor staining (Figures 4 and 11). This could have been due to differences in the depth of the scPVS from the surface of the abdominal subcutaneous tissue layer (Figures 4(b) and 4(c), two-way dotted white arrows and open arrows). Alternatively, it could also have been be due to variations in the individual rat's genetic or health states. In addition, the sinus structure (Figures 7(e) and 7(f)) in the cross sectioned slice of the scPVS vessel was confirmed twice during the 11th attempt. This could have been due to differences in the vessel structures and the morphological denaturalization during the tissue sectioning and staining. Further studies are needed to fully understand these variations and detailed experimental conditions.

In this study, we showed that the PVS is present on the subcutaneous tissue layer, which is likely to be target sites or area of acupuncture stimulations. It is first to show that the scPVS can be identified in a specific region of the body, namely, the abdominal midline in the subcutaneous tissue layer, and will help further study on the function of PVS in the body. For example, a selected part of a specific PVS could be damaged and then monitored for changes in physiological functions, and changes in the PVS in relation to changes in body states could be investigated [28], or the effect of specific acupuncture stimulation in relation to the changes in the selected PVS tissues could be monitored. In addition, this study will also provide a base for the study on the role of PVS in innate immunity since MCs, rich in the PVS, are important in innate immune system phenotypic and function [29].

## 5. Conclusion

In this study, the presence of scPVS in abdominal subcutaneous tissue has been identified for the first time using the Hemacolor staining technique. The scPVS has major features in common with the well-established PVS tissue (i.e., osPVS) in terms of gross morphology and cellular/structural characteristics. The scPVS present on the midline of the abdominal subcutaneous tissue layer was also found to overlap with the CV meridian and its acupoints. This study is the first to show the presence of the PVS on the superficial tissues of the body. These findings may help to identify other scPVS tissues in the body and further elucidate their pathophysiological roles in healthy and disease states.
